# Do Maternal Vitamin D Levels Influence Vitamin D Levels in Preterm Neonates?

**DOI:** 10.1155/2019/8613414

**Published:** 2019-01-01

**Authors:** M. Panda, J. McIntosh, T. Chaudhari, A. L. Kent

**Affiliations:** ^1^Dept of Neonatology, Centenary Hospital for Women and Children, Canberra Hospital, Woden, ACT, Australia; ^2^Dept of Neonatology, John Hunter Hospital, Newcastle, NSW, Australia; ^3^Australian National University Medical School, Canberra, ACT, Australia

## Abstract

**Objective:**

To determine the prevalence of Vitamin D (VitD) deficiency/insufficiency in mothers of preterm neonates less than or equal to 32 weeks of gestation and determine if the current level of VitD supplementation used for preterm neonates is appropriate.

**Design:**

Prospective study from 10^th^ May 2015 to 1^st^ November 2016.

**Setting:**

Neonatal Intensive Care Unit at the Canberra Hospital.

**Patients:**

Mothers and their preterm neonates born less than or equal to 32 weeks gestation.

**Interventions:**

Maternal VitD levels were obtained within 3-4 days following delivery. Neonatal VitD levels were obtained in the first 3-4 days of life, at 3-4 weeks of age, and at 6-8 weeks of age. Demographic data and data on VitD intake from parenteral nutrition, enteral feeds, and vitamin supplementation agents were collected.

**Results:**

70 neonates were enrolled into the study. Median gestation was 29 (27-30) weeks and median birth weight 1197 (971.2-1512.5) grams. Median maternal VitD level was 54.5 (36-70.7) nmol/L, median neonatal Vit D level at birth was 57 (42-70) nmol/L. Median Vit D level at 3 weeks and 6 weeks were 63.5 nmol/L (53-80.2) nmol/L and 103 (71.5-144) nmol/L respectively. 22/55 (40%) mothers were VitD deficient/insufficient. 25/70 (36%) neonates were VitD deficient/insufficient at birth. Of those neonates who were VitD deficient/insufficient at birth 5/25(10%) were deficient/insufficient at 6 weeks. The median intake of VitD at 6 weeks was 826.5 (577.5-939.5) IU/day.

**Conclusions:**

VitD deficiency/insufficiency in mothers of preterm neonates and in preterm neonates at birth is common. Routine screening of maternal VitD and their preterm neonates along with individualized supplementation regimens in mothers and preterm infants may optimize VitD status and reduce risk of ongoing VitD deficiency/insufficiency.

## 1. Introduction

Vitamin D (VitD) deficiency has significant health impacts particularly related to calcium and bone metabolism. Significant calcium deposition in the fetal skeleton occurs in utero during the third trimester; thus preterm infants are at risk of osteopenia [1], which can predispose to fractures [2]. Approximately 10-20% of preterm infants <1000g have radiographically defined rickets [3]. Optimizing calcium and phosphorus intake should prevent osteopenia of prematurity in majority of cases [4] but requires sufficient levels of VitD. In addition to bone and calcium related issues resulting from severe deficiency, VitD deficiency has also been linked to an increased risk of diabetes mellitus, certain cancers, and autoimmune diseases [5]. VitD receptors thus are thought to have multiple functions, many of which have not yet been fully elucidated, including roles in regulating genes that are involved in cell growth, immune function, and cardiovascular health. Given the widespread biologic effects of VitD it is reasonable to postulate that VitD deficiency in humans during the fetal and neonatal period may have long lasting health consequences [6].

Maternal Vit D deficiency has been shown to be detrimental to the fetus with increased risk of intrauterine growth restriction, preeclampsia, gestational diabetes mellitus, and preterm birth, all of which can have significant health impacts on the fetus and neonate [7, 8]. Studies have also shown that low cord blood levels of VitD were associated with an increased risk of respiratory infection during the first few months of life and increased risk of recurrent wheeze in early childhood [9]. Animal studies have demonstrated that VitD is likely to be important in lung development, with offspring of deficient mothers having reduced lung volumes and function [10]; the importance of this for preterm neonates is paramount given their already high risk of chronic lung disease. The potential long-term public health impact of preventing VitD deficiency, particularly during this critical time, is becoming increasingly evident.

Studies have shown 11-19% of neonates in Australia and New Zealand have levels <25nmol/L [11, 12]. A New Zealand study showed that 46% of pregnant women had Vitamin D levels <50nmol/l [13]. Studies also show that up to 18% of pregnant women in predominantly Caucasian populations had VitD levels <50nmol/L [11, 14, 15]. However, the implications to the fetus of maternal VitD deficiency are not yet fully understood and more research is required.

There is no worldwide consensus on the recommended daily requirements for VitD for preterm infants. In the United States a recommended daily intake for VLBW infants is 200 to 400IU [16] and the European Society for Paediatrics Gastroenterology, Hepatology, and Nutrition recommends 800-1000 IU [17]. Studies in preterm neonates have shown that supplementation with amounts from 400- 3000 IU/day is safe. Several studies have shown that supplementing with standard levels of VitD may be insufficient for some preterm neonates [12, 18].

The aims of this study were to determine (1) the prevalence of VitD deficiency in mothers of preterm neonates and (2) if the current level of VitD supplementation is appropriate.

## 2. Methods

This is a prospective cohort study carried out in a single Neonatal Intensive Care Unit (NICU) at The Centenary Hospital for Women and Children, ACT from 10^th^ May 2015 to 1^st^ November 2016.

Inclusion criteria were mothers and their preterm neonates born less than or equal to 32 weeks gestation and admitted to the NICU. Neonates were excluded if they had significant chromosomal or congenital anomalies. Mothers and their babies were recruited and consented in the first 72 hours postdelivery. Maternal data collected included whether there was antenatal VitD supplementation, ethnicity and season of birth. Standard variables including sex, gestational age, and weight were recorded.

Maternal 25(OH) D_3_ was measured in the immediate postnatal period (day 1-4). Enrolled neonates had their 25(OH) D_3_ levels measured during the first 3 days of life, at 3-4 weeks, and 6-8 weeks of age. Neonatal serum calcium, phosphorus, and alkaline phosphatase were collected at the time of VitD collection. 25(OH) D_3_ was measured using the Liaison ® 25 OH vitamin D immunoassay. The definitions of VitD deficiency used for this study were deficiency <30nmol/L, insufficiency 30-49nmol/L, and sufficiency ≥ 50nmol/L.

Neonates were supplemented with VitD as per departmental guidelines - 160IU/day when on TPN with lipids; 800 IU/day from 7 days or when on full feeds from Pentavite; 120IU/100ml preterm formula; 120IU/100ml human milk fortification and 25IU/L in maternal breast milk. The daily amount of VitD supplementation was estimated for the first 8 weeks of life from neonatal fluid and medication charts. This was estimated from the combined intake of pentavite (400IU/0.45ml), TPN with lipids, formula, human milk fortifier, and breast milk. The amount received by each preterm neonate varied throughout their NICU stay and depended on feeding regimen and fluid intake.

During the early part of the study period (after 14 patients had had their 6-8 weeks VitD level measured) it was noted that 50% of neonates had VitD levels >150nmol/L, with an average level of 152nmol/L for all subjects at that time point. While there had been no evidence of adverse effects of high VitD levels, the routine supplementation dose of pentavite was reduced from 800 IU to 400 IU daily.

Data were stored and analysed using IBM SPSS statistics version 22 (SPSS: An IBM Company. Chicago Ill, USA, 2010). Data will be presented as number and percentage with odds ratio and 95% confidence interval (CI) or median and interquartile ranges.

This study was approved by the ACT Health Human Research Ethics Committee.

## 3. Results

Seventy premature neonates were enrolled in the study. The median (IQR) gestational age was 29 (27-30) weeks and birth weight was 1197.5 (971.2-1512.5) grams. Majority of neonates (70%) were enrolled during the autumn-winter period and Caucasians formed the major ethnic group comprising 71.4% of the cohort with 8.5% of neonates being of Aboriginal descent. Demographic characteristics are shown in Table 1.

### 3.1. Maternal Vitamin D Status

Of the 70 neonates enrolled in the study, maternal serum VitD level in the immediate postnatal period was available for 55 mothers, therefore only these were included for analysis of VitD status in mothers. The median (IQR) VitD level at delivery was 54.5 (36-70.7) nmol/L. VitD deficiency was noted in 18.2% of mothers, 21.8% had VitD insufficiency, and 60% had sufficient VitD levels (Table 2). Thirty (55%) of the 55 mothers were on VitD supplementation. The prevalence of Vit D insufficiency/deficiency in women receiving supplementation was 30%. The percentage insufficient/ deficient was higher at 52% in the nonsupplemented group (p=0.11). The levels of Vit D in mothers who received supplementation was 58.0 (22-176) nmol/L which was significantly higher than mothers who did not receive supplementation, 41.0 (20-86) nmol/L, (p=0.04). The prevalence of VitD insufficiency/deficiency amongst Caucasian mothers was 34% as compared to 53% in non-Caucasian mothers (p=0.24). Fifty-three percent of non-Caucasian mothers received VitD supplementation compared to 55% Caucasian mothers (p=1.0).

### 3.2. Vitamin D and Bone Markers in Preterm Neonates

**Figure 1:**
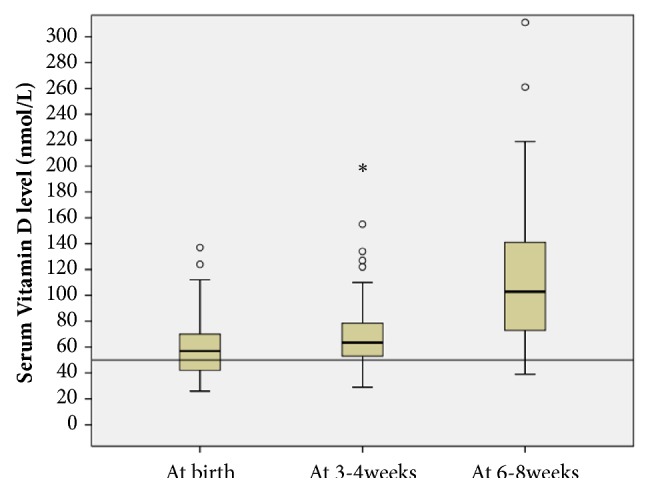
Serum Vitamin D (25 (OH) D_3_) levels of preterm neonates at different time frames. Values of serum vitamin D are expressed in nmol/L.

25 (OH) D_3_ and bone markers were available for all 70 neonates within the first 3 days of life. This data was available for 60 neonates at 3-4 weeks and for 49 neonates at 6-8 weeks. The decrease in available results was predominantly due to transfer to other hospitals for convalescent care. The median (IQR) VitD level within the first 3 days of life was 57 (42-70) nmol/L, which increased to 63.5 (53-80.2) nmol/L between 3 and 4 weeks and to 103 (71.5-144) nmol/L between 6 and 8 weeks (Figure 1). Serum levels of VitD and bone markers at these three-time frames are shown in Table 3.

The prevalence of VitD insufficiency at birth was 30%; which decreased to 15% at 3-4 weeks and to 10.2% at 6-8 weeks (p=0.001). Also, a reduction in the prevalence of VitD deficiency was seen from 5.7% at birth to 1.7% at 3-4 weeks to none at 6-8 weeks (Table 4). Overall, the combined prevalence of VitD insufficiency/deficiency at birth was 36%, which decreased to 17% at 3-4 weeks (p=0.02) and further to 10% at 6-8weeks (p=0.002). All of the neonates who were insufficient at 6-8 weeks were insufficient/deficient at birth. The number of babies who had sufficient VitD levels was significantly higher at 3-4 weeks (p-=0.02) and 6-8 weeks (p=0.002) compared to birth levels. There was no significant difference in sufficiency at 3-4 weeks compared to 6-8 weeks (p=0.41). There was a positive correlation between VitD levels at birth and 3-4weeks (r=0.374, p=0.003) but not between birth and 6-8 weeks (r=0.248, p=0.086). There was a strong correlation between VitD levels at 3-4 weeks and 6-8 weeks (r=0.632, p<0.001).

**Figure 2:**
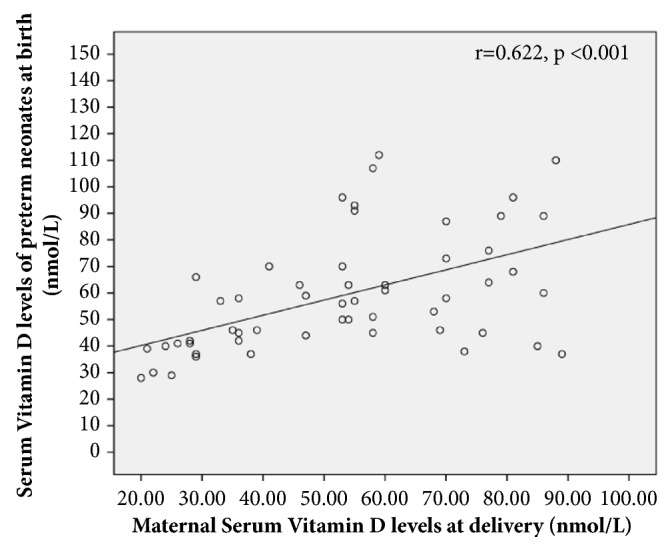
Correlation of Maternal Vitamin D levels and Preterm Vitamin D levels at birth.

Low serum maternal VitD was associated with a higher likelihood of VitD insufficiency/deficiency in neonates at birth (p<0.001) and at 3-4 weeks (p=0.006) but not at 6-8 weeks (p=0.14). The prevalence of VitD insufficiency/deficiency in preterm infants whose mothers did not receive VitD supplementation was 44% as opposed to 37% in infants whose mothers received Vit D supplementation (p=0.60). Thus, there was no difference in neonatal birth VitD levels in relation to maternal supplementation during pregnancy alone. However, there was a strong positive correlation between maternal VitD levels and preterm VitD levels at birth, r=0.622 (Figure 2).

Neonates of non-Caucasian descent were more likely to be VitD insufficiency/deficiency compared to Caucasian neonates (65% versus 24% (p=0.002)). However, no association was observed between VitD status and sex, season of birth, gestational age, and birth weight. Comparison of incidence of sepsis and necrotizing enterocolitis with VitD status at birth also showed no significance (Table 5).

Subgroup analysis comparing the prevalence of VitD deficiency amongst premature neonates receiving high dose pentavite (800 IU/day of VitD) against those receiving low dose pentavite (400 IU/day of VitD) showed that, at 6-8 weeks, the prevalence of VitD insufficiency was 7.1% in the high dose group as compared to 11.4% in the low dose group (p=0.65). The median serum VitD level in the high dose group at 3-4 weeks was 61(49.5-100.5) nmol/L, similar to that in the low dose group 64 (53.7-76.7) nmol/L. However, at 6-8 weeks, the high dose group had a higher VitD level [126 (69.2-182.2) nmol/L] compared to the low dose group [96 (73-131) nmol/L], p=0.25. The differences between serum bone markers-calcium, phosphate, and alkaline phosphatase were not statistically different in the high and low dose group (Table 6).

### 3.3. Vitamin D Intake in Preterm Neonates

The majority of preterm neonates between 3-4 weeks and 6-8 weeks of age were breast fed, 60% (n=42) and 57.1% (n=44) respectively. The median VitD intake at 3-4 weeks increased from 749.5 (554.2-861) IU/day to 826.5 (577.5-939.5) IU/day at 6-8 weeks (p=0.06). At 3-4weeks the median VitD intake in sufficient group was 761.5 (604.5-866.7) IU/day which was significantly higher as compared to insufficient/deficient group, 618 (197.2-755.7) IU/day (p=0.04). However, at 6-8weeks the median VitD intake in sufficient group as compared to insufficient/deficient group was not statistically significant, p=0.22.

## 4. Discussion

This study shows that maternal VitD status has implications on VitD status of preterm neonates at birth and at 3-4 weeks post birth. Our study indicates that current dosing regimens of VitD may not be adequate for some neonates and that VitD levels should be performed on all preterm neonates at 3-4 weeks of life. In addition, VitD levels at birth correlated with those at 3-4 weeks. Hence, we suggest that VitD levels should be performed in all high risk VLBW neonates - those born to non-Caucasians or to mothers with VitD deficiency. Our recommendation of targeting a higher dose of VitD in deficient babies is similar to that suggested by Fort et al [21].

In our cohort maternal VitD level was insufficient/deficient in 40% of mothers, despite the fact that 55% of the mothers were supplemented with VitD. This was slightly higher than in the population study done by Perampalam et al. where 35% of pregnant women had 25(OH) D_3_ less than 50nmol/L [22]. This may be due to the fact that majority of our cohort were enrolled in winter/spring. In our study, maternal VitD supplementation did not have a significant influence in reducing maternal VitD insufficiency/deficiency as well as in preterm infant VitD status. This can be explained by the fact that the amount of maternal VitD supplementation and compliance to treatment were unknown. Our study showed that low maternal VitD level was associated with significantly higher deficiency in neonates at birth and at 3-4 weeks. There was positive correlation between maternal VitD levels at birth and preterm VitD at birth. Similar findings have also been reported in previous studies [12, 13, 23]. The prevalence of VitD deficiency in preterm infants was also higher in the non-Caucasian population in our cohort (p=0.003), similar to the findings of Monangi et al. [12]. In non-Caucasians melanin decreases the amount of ultraviolet B radiation reaching the basal skin layer to generate the production of cholecalciferol (Vitamin D_3_) [24]. Metanalysis done by Qin et al found that pregnant women with VitD levels <50nmol/L experienced a significantly increased risk of preterm birth [25]. The Australian clinical practice guideline on antenatal care does not currently recommend routine screening of VitD levels; however, risk-based screening is common in clinical practice.

Epidemiological surveys from Australia and New Zealand have shown that 40-57% of neonates have VitD levels <50nmol/L [9, 11]. The prevalence of VitD insufficiency/deficiency in our study at birth was 35.7%; which decreased to 7.1% at the time of discharge with our current supplementation regimen. There is no clear consensus at present as to what exact level of vitamin D constitutes sufficiency in terms of biologic and health effects at any age, particularly neonatal, and ranges from 50-80nmol/L are most often quoted [26, 27]. There was positive correlation between VitD levels at birth and at 3-4weeks. Previous research has demonstrated that VitD levels in preterm or low birth weight babies rise rapidly during the first 1-2 weeks of VitD supplementation (with 1000-3000IU) and then plateau around 82nmol/ml after 7 weeks, suggesting that this is a healthy neonatal level [1, 28]. Our data was in agreement to this and the median level of VitD increased from 57nmol/L during the first week to 103nmol/L between 6 and 8 weeks.

At the beginning of our study, high levels of VitD (>150nmol/L) as well as hypercalcemia (serum calcium > 2.5mmol/L) was observed in 50% of neonates (7 out of 14 babies) at 6-8 weeks. In view of these results, pentavite supplementation was ceased in all 7 neonates. All these neonates were receiving VitD supplementation at 800IU/day. The amount of breast milk fortification, calcium and phosphate supplementation remained the same both during 800IU/day and 400 IU/day of Vit D supplementation as per our unit protocol. Furthermore, median phosphate and ALKP levels were similar in both the groups suggesting hypercalcemia was due to high Vit D levels rather than hypophosphatemia. High VitD levels can cause increased calcium absorption from the intestines resulting in hypercalcemia and hypercalciuria leading to polyuria, dehydration, poor growth, and extra-skeletal calcification especially nephrocalcinosis. Therefore, reviewing VitD levels in preterm neonates with hypercalcemia on VitD supplementation can become important to guide supplementation dosage. The calcium levels normalized in all 7 babies following cessation of pentavite. As such, we did not perform renal ultrasound prior to discharge for nephrocalcinosis. Levels of serum VitD being high at 6-8 weeks have been reported in studies by Fort et al. [19] and Tergestina et al. [29]. There was no significant increase in Vit D deficiency in our cohort following dose reduction; which might mean that a daily supplementation of 400IU/day should be adequate to obtain a sufficient vitamin D level. Fort et al. suggested that higher dosing for the first 1-2 weeks of life to get VitD levels into the normal range followed by lower dosages may be more appropriate to prevent high serum levels of VitD occurring [21]. However, no adverse effects were seen in a trial with higher neonatal VitD levels [29].

VitD status of preterm infants is entirely dependent on extrinsic supplementation as VitD synthesis from sunlight is lacking. The recommendations for VitD supplementation vary widely between different NICUs' from 400IU to 1000IU [8, 9] and the current trend of fortification of breast milk provides additional VitD depending on individual unit protocol. Most of our neonates were receiving fortified breast milk which contains calcium as well as phosphate along with additional VitD supplementation in the form of Pentavite. The median VitD intake in our preterm cohort increased following supplementation from 749.5 (554.2-861) IU/day at 3-4 weeks to 826.5(577.5-939.5) IU/day at 6-8 weeks. The lower VitD intake in the high dose group at 6-8 weeks as compared to low dose group was secondary to cessation of VitD supplementation following high serum levels (>150nmol/L) of VitD at 3-4 weeks, thus emphasizing that perhaps screening at 3-4 weeks should be done so as to adjust VitD dosage.

The limitation of our study was that it was not powered to assess clinical outcomes such as sepsis, necrotizing enterocolitis, chronic lung disease, and growth and developmental outcomes. The amount of maternal supplementation of VitD was not documented as this was not part of our study protocol. Also, there was a 30 percent attrition rate in our sample size from enrollment at birth to that at 6-8 weeks secondary to inter hospital transfer.

To our knowledge, this study is the first showing a trajectory of vitamin D status at three different time frames in extremely preterm neonates. These results are generalizable to other NICUs, as the cohort of patients is similar to other neonatal intensive care units across Australia and New Zealand, with similar VitD supplementation regimens in parenteral nutrition, vitamin supplementation, and fortification of feeds. Given the significant proportion of preterm neonates that are deficient/insufficient at birth with a proportion still being deficient/insufficient at 3-4 weeks of life, there is a potential role for VitD screening of all preterm neonates at 3-4 weeks of life to ensure adequate VitD supplementation.

## 5. Conclusion 

This study shows that VitD deficiency/insufficiency is common in mothers of preterm neonates and consequently in preterm neonates at birth. Our current supplementation practices ensure that the majority of neonates have sufficient VitD levels at 6-8 weeks of life; however VitD supplementation in mothers and postnatally in preterm infants needs to be individualized so as to normalize the serum VitD level in the mothers during the third trimester as well as in the preterm infants from the first few days of life.

## Figures and Tables

**Table 1:** Demographic characteristics.

Characteristics	All neonates (n=70)
Male n (%)	50 (71.4)

Gestational age, weeks^*∗*^	29(27-30)

Birth weight in grams^*∗*^	1197.5(971.2-1512.5)

Ethnicity, n (%)	
Caucasian	50(71.4)
Aboriginal	6(8.5)
African	4(5.7)
Eurasian	3(4.3)
South American	3(4.3)
Indian	2(2.9)
Asian	2(2.9)

Season at birth, n (%)	
Summer-spring	21(30)
Autumn-Winter	49(70)

^*∗*^Values are median (IQR).

**Table 2:** Maternal vitamin D status in the immediate postnatal period.

	Number (Total 55)	Percentage (%)
Sufficient (≥50nmol/L)	33	60

Insufficiency (30-49nmol/L)	12	21.8

Deficiency (<30nmol/L)	10	18.2

**Table 3:** Serum bone markers in preterm neonates.

	1-3 days	3-4 weeks	6-8 weeks
25 (OH) D_3,_nmol/L	57 (42-70)	63.5 (53-80.2)	103 (71.5-144)

Calcium, mmol/L	2.42 (2.16-2.53)	2.72 (2.63-2.77)	2.68 (2.63-2.73)

Phosphate, mmol/L	1.90 (1.63-2.17)	2.15 (1.86-2.29)	2.10 (1.87-2.29)

ALKP, IU/L	219 (164-268)	331 (270-439)	324 (256-376)

Values are expressed as median (IQR). ALKP: alkaline phosphatase.

**Table 4:** Vitamin D deficiency status at different time intervals.

	1-3 days	3-4 weeks	6-8 weeks	P Value
	N=70 (%)	N=60 (%)	N=49 (%)	
Sufficiency	45 (64.3)	50(83.3)	44(89.8)	0.667
(≥50nmol/L)				

Insufficiency	21 (30.0)	9(15)	5(10.2)	**0.001**
(30-49nmol/L)				

Deficiency	4(5.7)	1(1.7)	-	0.18
(<30nmol/L)				

**Table 5:** Factors associated with vitamin D status at different time points in preterm infants.

**Variable**	**Vitamin D insufficiency/deficiency (p value)**
**Low serum maternal vit D**	**P<0.001** ^**∗**^
**(<50nmol/L)**	**P=0.006** ^**#**^

**Ethnicity**	**0.003##**

**Season-Winter/Autumn**	**0.954**

**GA**	**0.382** ^**∗**^

**Birth weight**	**0.646** ^**∗**^

**Male sex**	**0.986** ^**∗**^

**Sepsis **	**0.500** ^**∗**^

**NEC**	**0.838** ^**∗**^

^*∗*^p value at birth, #p value at 3-4 weeks, ##non-Caucasian. Sepsis definition (CDC definition): a neonate was considered to have a BSI if there was (1) a definite pathogen in blood culture OR (2) growth of a possible contaminant (e.g., coagulase-negative staphylococcus, CONS) in blood PLUS treatment with antibiotics ≥96h (or death <96h) PLUS. Growth of the same organism on repeat culture OR one or more abnormal laboratory markers (e.g., C-reactive protein >10mg/L, immature: total neutrophil ratio >0.2) (definite infection) OR clinical features consistent with systemic infection (e.g., lethargy, apnoea, and significant change in respiratory condition) (clinical infection) [19, 20].

**Table 6:** Serum bone markers in high dose group and low dose group at 6-8weeks.

	High dose group	Low dose group	P value
	N=14	N=35	
25 (OH) D_3,_nmol/L	126(69.2-182.2)	96 (73-131)	0.25

Calcium, mmol/L	2.68 (2.5-3.0)	2.68(2.3-2.9)	0.99

Phosphate, mmol/L	2.05(1.4-2.2)	2.13(1.0-2.5)	0.46

ALKP, IU/L	328(211-851)	310 (132-853)	0.33

Values are expressed as median (IQR). ALKP: alkaline phosphatase.

## Data Availability

Raw data for this study was collected prospectively using data pro forma approved by the institutional human research ethics committee (approval number eth.1.15.002). This data are available from the corresponding author upon reasonable request.
